# BoHV-4 immediate early 1 gene is a dispensable gene and its product is not a bone marrow stromal cell antigen 2 counteracting factor

**DOI:** 10.1186/s12917-015-0540-4

**Published:** 2015-08-27

**Authors:** Valentina Franceschi, Antonio Capocefalo, Sarah Jacca, Alfonso Rosamilia, Sandro Cavirani, Fengwen Xu, Wentao Qiao, Gaetano Donofrio

**Affiliations:** Department of Medical-Veterinary Science, University of Parma, via del Taglio 10, 43126 Parma, Italy; Key Laboratory of Molecular Microbiology and Biotechnology, College of Life Sciences, Nankai University, Tianjin, China

**Keywords:** Bovine herpesvirus 4, Viral vector, Bacteria artificial chromosome, Immediate early gene, Gene knock-down, Impaired viral replication

## Abstract

**Background:**

Bovine herpesvirus 4 (BoHV-4) is a gammaherpesvirus whose genome was cloned as Bacterial Artificial Chromosome (BAC) and exploited as a gene delivery vector for vaccine purposes. Although BoHV-4 genome has been completely sequenced and its open reading frames (ORFs) structurally defined *in silico*, most of them are not functionally characterized. In BoHV-4 genome two major immediate early genes (IE) are present, IE1 and IE2. IE2 is an essential gene because its removal from the viral genome renders the virus unable to replicate, whereas for IE1 no many functional information are available.

**Results:**

In this work, IE1 contribution in initiating and maintaining BoHV-4 lytic replication was assessed generating a recombinant BoHV-4 genome lacking of IE1 gene, BoHV-4ΔIE1. In contrast to BoHV-4IE2 deleted mutant, BoHV-4ΔIE1 infectious replicating viral particles (IRVPs) could be reconstituted following viral DNA electroporation in permissive cells. However the titer of BoHV-4ΔIE1 IRVPs produced into the cell supernatant and BoHV-4ΔIE1 plaques size were reduced respect to BoHV-4 undeleted control. Further the impaired BoHV-4ΔIE1 IRVPs produced into the cell supernatant could be rescued by expressing IE1 gene product *in trans*, confirming the implication of IE1 in BoHV-4 lytic replication. Next, the possible role of BoHV-4IE1 as bone marrow stromal cell antigen 2 (BST-2) counteracting factor, as hypothesized by IE1 amino-terminal gene product homology with Kaposi Sarcoma Associated Herpesvirus (KSHV) K5, was excluded too.

**Conclusions:**

Although the real function of BoHV-4IE1 is still elusive, a new BoHV-4 genome gene locus as a target site for the insertion of foreign DNA and resulting in the attenuation of the virus has been revealed. These data can be considered of relevance to improve BoHV-4 gene delivery properties.

**Electronic supplementary material:**

The online version of this article (doi:10.1186/s12917-015-0540-4) contains supplementary material, which is available to authorized users.

## Background

Bovine herpesvirus 4 (BoHV-4) is a gammaherpesvirus of the *genus* Rhadinovirus, and has been isolated both in healthy animals and in animals with variable diseases, from ocular discharge, conjunctivitis, dermatitis, respiratory diseases and abortion [1–3]. The pathogenic role of BoHV-4 remains unclear, even if there is increasing evidence of a secondary pathogenic role in bovine post-partum metritis [3–8]. The coding capacity of BoHV-4 has been described as well as the common and unique features of the BoHV-4 genome in comparison to those of other gammaherpesviruses. However many of BoHV-4 genes and their products have not been functionally characterized [9].

BoHV-4 gene expression cascade is similar to that of other herpesviruses and comprises Immediate Early (IE), Early (E) and Late (L) gene expression [10]. Herpesvirus IE genes are experimentally defined as those which are transcribed when cells are infected in the presence of protein synthesis inhibitor, because IE gene expression does not require *de novo* viral protein synthesis. Under this conditions, RNA transcribed from IE genes usually accumulates to higher levels than in absence of inhibitors, presumably because of the lack of feed-back inhibition. Two major BoHV-4IE RNAs were characterized early during infection in the presence of cycloheximide, IE1 and IE2 [11]. Although both of them have been well characterized in terms of gene structure, transcription and RNA post-transcriptional processing [11, 12], the only one to be functionally characterized was IE2 [13, 14]. The generation of viral mutants targeting the IE2 locus within the BoHV-4 genome, provided the direct demonstration that BoHV-4 gene product, ORF50/*Rta*, is indispensable in initiating and allowing progression of BoHV-4 lytic replication [13, 14]. In fact a recombinant BoHV-4 in which ORF50/*Rta* was deleted was completely unable to replicate, but was efficiently rescued, with respect to production of infectious virus and DNA replication, upon the expression of ORF50/*Rta in trans* [14]. Whereas as regards BoHV-4IE1 gene, it is the most abundant viral RNA transcribed in the presence of cycloheximide, though its abundance is greatly reduced in absence of inhibitor, suggesting a down regulation by newly synthesized viral proteins [11]. Since BoHV-4IE1 RNA is the major RNA found under IE conditions, it was referred to it as the major IE RNA [11]. IE1 is a spliced, 1,7 kb RNA, containing four exons and transcribed from the right to the left of BoHV-4 genome. IE1 open reading frame (ORF) codes for a protein of 285 amino acids (aa) with a predicted molecular weight of 33 kDa and an unknown function [11]. Therefore, the purpose of the present work was to knock-down BoHV-4IE1 gene in BoHV-4 genome cloned as a BAC, to reveal its potential contribution in initiating and maintaining BoHV-4 lytic replication.

## Results and discussion

### Generation of a BoHV-4IE1 deleted mutant

**Fig. 1:**
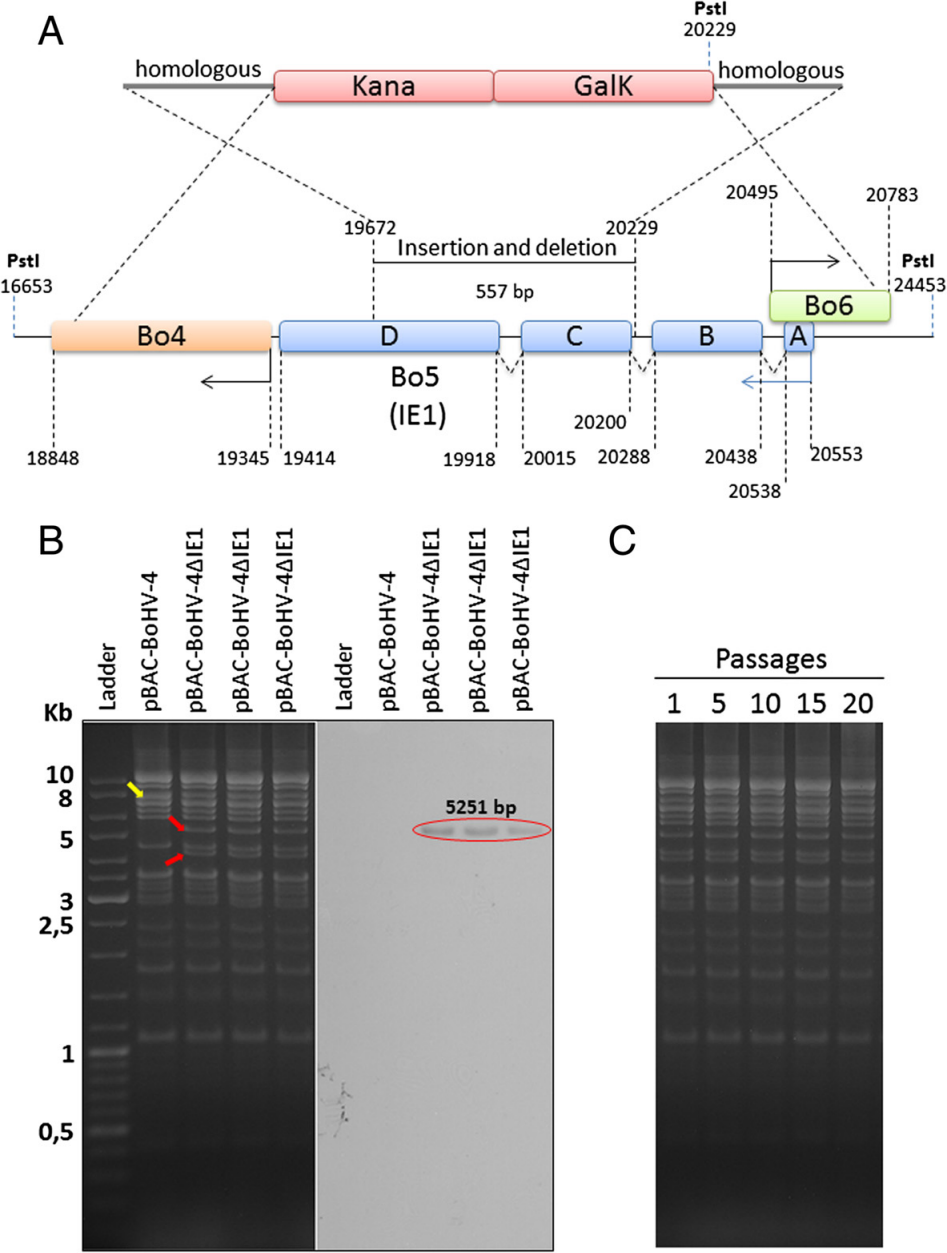
Generation of BoHV-4-ΔIE1. **a** Schematic representation (not on scale) of the overall strategy to knock-down IE1 gene in BoHV-4 genome clone in SW 102 *E. coli*. Bo4 (*orange*), Bo5 [(IE1), *blue*] and Bo6 (*green*) gene coding regions are indicated by numbers, which represent nucleotide positions within the BoHV-4 genome [based on the BoHV-4-66p347 complete genome published sequence (GenBank accession number AF318573)]. The targeting fragment (*red*), IE1L-KanaGalK-IE1R, flanked by two homologous sequences allowed the insertion of the double selectable marker KanaGalK between the positions 19,672 and 20,229, deleting most of the IE1 coding region but leaving intact the Bo4 and Bo6 promoters. IE1 locus possess two PstI sites, one at the position 16,653 and one at the position 24,453, generating a fragment of 7800 bp. After targeting, a new PstI site, delivered by the targeted vector, was introduced into the IE1 locus and thus generating three PstI restriction sites and two PstI digestion product, of 4224 and 5251 bp respectively. PstI restriction enzyme digestion allowed to distinguish between targeted and untargeted clones (**b**), in fact the targeted clones (pBAC-BoHV-4ΔIE1) display two new bands (*indicated by red arrows*) respect to the untargeted control clone (pBAC-BoHV-4; *indicated by the yellow arrow*). This is further confirmed by southern blotting with a specific probe for KanaGalK (*red circle*). **c** Clonal stability of pBAC-BoHV-4ΔIE1 in SW 102 *E. coli* at passage 1, 5, 10, 15, 20 and analyzed by PstI restriction digestion

Although BoHV-4IE1 is simultaneously expressed along with IE2 during BoHV-4 life cycle and IE2 has a pivotal role in initiating the BoHV-4 transcriptional replication [13, 14], it was of interest to know if IE1 could have an essential role similarly to that observed for IE2. The main way to achieve this kind of information was to knock-down IE1 gene coding regions by heat inducible homologous recombination into the genome of BoHV-4 cloned as bacterial artificial chromosome. A targeting fragment, IE1L-KanaGalK-IE1R, containing the 2232 base-pairs (bp) KanaGalK double selecting cassette [15] flanked by two BoHV-4IE1 gene homologous sequences, was introduced between the BoHV-4 genome position 19,672 and 20,229. This insertion, comprising the full deletion of the IE1 third exon and most of the fourth exon, resulted with the elimination of the 70 % of the IE1 coding regions. Many genes of BoHV-4 genome are overlapped and the coding region of a gene can also works as a regulatory region for the neighboring gene. This IE1 gene knock-down strategy allowed maintaining intact the Bo4 and Bo6 gene promoter, thus preserving their transcription and translation (data not shown). Therefore, the viral phenotype obtained from this insertion/deletion was exclusively due to BoHV-4IE1 knock-down and not contaminated by the loss of expression of the flanking genes, in this specific case Bo4 and Bo6 (Fig. 1a). To generate BoHV-4 with knocked-down IE1 gene, BoHV-4ΔIE1, linearized pIE1L-KanaGalK-IE1R was electroporated in SW102 *E. coli* containing pBAC-BoHV-4 genome and pBAC-BoHV-4ΔIE1 was generated. The authenticity of the selected targeted clones were checked by PstI restriction enzyme digestion and confirmed by southern hybridization by a specific probe (Fig. 1b). Further, pBAC-BoHV-4ΔIE1 clone stability in SW 102 *E. coli*, before to attempt the reconstitution of the IE1 deleted virus, was assessed by serial passages (over 20) and PstI digestion. pBAC-BoHV-4ΔIE1 was stable as observed by the absence of differences among the restriction profile of pBAC-BoHV-4ΔIE1 derived from different passages (Fig. 1c).

### BoHV-4ΔIE1 has an impaired replication

**Fig. 2:**
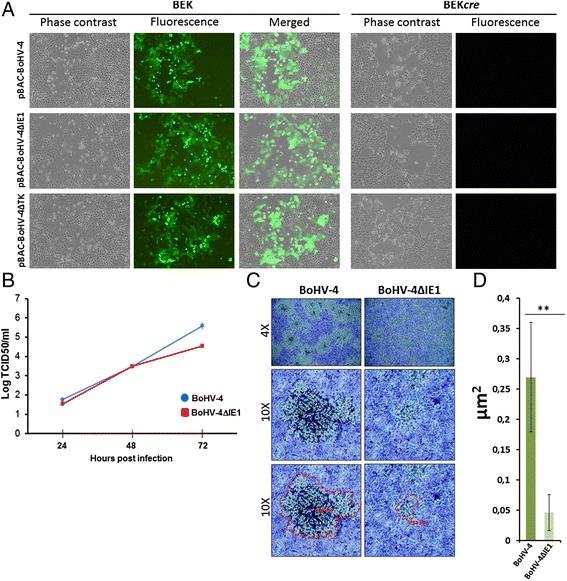
Characterization of BoHV-4-ΔIE1. **a** Representative images (phase contrast, fluorescence and merged; 10×) of BEK and BEK expressing *cre* (BEK*cre; in the right panel*) electroporated with pBAC-BoHV-4 and pBAC-BoHV-4ΔIE1 DNA. CPE induced by reconstitution of IRVPs is recognizable for all three BAC viral genomes (dark pictures in fluorescence for BAC transfected BEK*cre* cells is due the loss of GFP expression cassette contained in the floxed BAC backbone removed by *cre* recombinase). The test was repeated three times always giving identical results. **b** Replication kinetics of BoHV-4ΔIE1 (*red line*) and BoHV-4 (*blue line*). **c** Representative images (4× and 10×) showing plaque morphology and relative plaque sizes of BoHV-4 and BoHV-4ΔIE1 on Vero cells. **d** The plaque sizes (μm^2^) were measured using the Axio-Vision40-V4.6.3.0 (Carl Zeiss, Imaging Solution) software program. Bars represent means ± standard errors of 50 plaques for each virus [(**) P ≤ 0.001)]. Significance was measured by ANOVA

Herpesvirus gene expression cascade and replication is mainly governed by the immediate early gene expression. The competence of the viral DNA genome to reconstitute Infectious Replicating Viral Particles, IRVPs, can be tested by transfecting the mutant viral genome in permissive cell lines and this is greatly facilitated if the viral genome, as is the case of BoHV-4, is cloned as a BAC. As a first approach to test the characteristics of the viral phenotype obtained from IE1 knock-down, a viral reconstitution assay was employed. BEK cells and BEK*cre* cells, constitutively expressing *cre* recombinase [16] to excise out the floxed BAC cassette from the viral genome, were electroporated with pBAC-BoHV-4, pBAC-BoHV-4ΔIE1 and pBAC-BoHV-4ΔTK, a mutant BoHV-4 genome in which the 2232 bp KanaGalK double selectable marker was inserted into the BoHV-4 thymidine kinase (TK) gene [17–19] without interfering with the replication property of the resulting virus. Surprisingly, all three genomes could efficiently reconstitute IRVPs (Fig. 2a) and this was the first consisting indication that IE1 is not an essential gene contrarily to what it was observed for BoHV-4IE2 and gB Knock-down, which gave a complete replicating uncompetent phenotype [14, 18]. Although the viral reconstitution assay is very informative from the qualitative point of view, on the other hand it is poorly quantitative. Thus the replication kinetics of BoHV-4ΔIE1 and BoHV-4 obtained from pBAC-BoHV-4ΔIE1 and pBAC-BoHV-4 BEK*cre* transfected cells were compared. A slower replication, of ~1 log, for BoHV-4ΔIE1 respect to BoHV-4 was observed (Fig. 2b). Although IE genes are the first genes to be transcribed during the infection, their function can be protracted for the entire viral replication cycle. BoHV-4 is a Gammaherpesvirus with a slow replication cycle respect to alpha and beta herpesvirus and this is the reason why we see differences during the late infection. This data was corroborated by the reduction of the plaque size generated by BoHV-4ΔIE1 respect to BoHV-4 (Fig. 2c and d).

### BoHV-4-ΔIE1 impaired replication can be rescued by IE1 expressed in trans

**Fig. 3:**
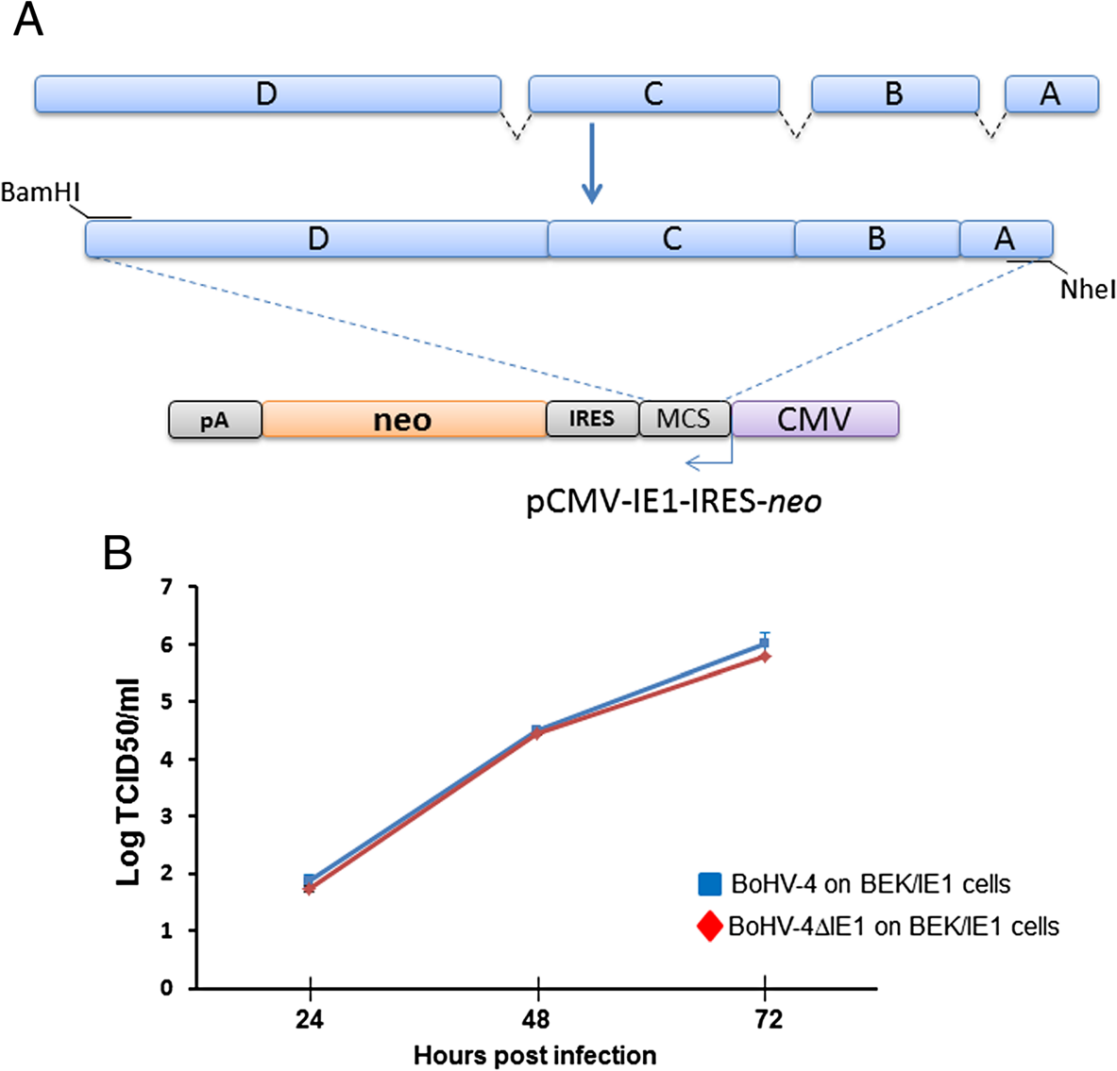
Rescue of BoHV-4-ΔIE1 impaired replication in trans. **a** Schematic diagram (not on scale) of BoHV-4IE1 gene containing exons (A, B, C and D) and introns (*dashed lines*), along with its reverse transcribed cDNA, amplified by PCR with a couple of primers containing restriction sites and to allow its subcloning into the bicistronic vector. **b** Replication kinetics of BoHV-4ΔIE1 (*red line*) and BoHV-4 (*blue line*) on BEK/IE1 cells

To confirm that the impaired replication, despite of small entity (~1 log) but significant, observed for BoHV-4ΔIE1, was due to the loss of IE1 and not a mere artifact, BEK cells constitutively expressing IE1 were generated to rescue *in trans* the loss of IE1. IE1 ORF cDNA was obtained by PCR with an antisense primer containing a restriction site, a kozak’s sequence and matching the first eight codons comprising the *atg*, and a sense primer matching the last eight codons, including the *stop* codon, end ending with a restriction site (Table 1; Fig. 3a). So generated IE1 ORF cDNA was sub cloned in a bicistronic expression vector provided of the CMV promoter, an internal ribosomal entry site (IRES) and a selectable marker (*neo*) (Fig. 3a). pCMV-IE1-IRES-*neo* was electroporated in BEK cells and cells selected with G418 to get BEK/IE1 cells. Next, BoHV-4ΔIE1 and BoHV-4 replication kinetics were compared on BEK/IE1 cells. As hoped, and showed in Fig. 3b, the gap of replication between BoHV-4ΔIE1 and BoHV-4 was filled by expressing IE1 *in trans*. Although a revertant strain could be generated, however it was reasoned that a revertant strain is not so much different from a wild type and for this reason it was preferred the expression in *trans* of the mutated gene.

**Table 1:** List of primers used in that work

Primer name	5′-3′ Primer sequence
IE1-left-sense	5′-CCC GAA TTC CAA TTG ACA ACA TAT AAA GTC-3′
IE1-left-antisense	5′-CCC GGT ACC CGA TTT GTC TTC ATT GCT GGT-3′
IE1-right-sense	5′-GGG CTG CAG AGC CAA AGA TGG CAT ATT GGG-3′
IE1-right antisense	5′-CCC AAG CTT CAA TTT CTT CAT TCC AAA CAC-3′
IE1-NheI-sense	5′-CCC GCT AGC CCA CC ATG GCC AGT AAA GAC T-5′
IE1-BamHI-anti	5′-CCC GGA TCC TCA TGT CCT GAG TGG GTC TAT GTT-3′
SmaI-AseI-Kana sense	5′- AAC CCC CGG GAT TAA TCC GGA ATT GCC AGC TGG GG-3′
SmaI-MluI-Kana anti	5′- CCA ACC CGG GAC GCG TGA AAT TGT AAG CGT TAA TAA T-3′
BST2sense-EcoRV	5′-CCC GAT ATC CCACC ATG GAT TAC AAG GAT GAC-3′
BST2anti-NheI	5′- CCC GCT AGC TCA GGT CAG CAG AGC GTT GAG GAC-3′

### BoHV-4IE1 is not a tethering counteracting factor

**Fig. 4:**
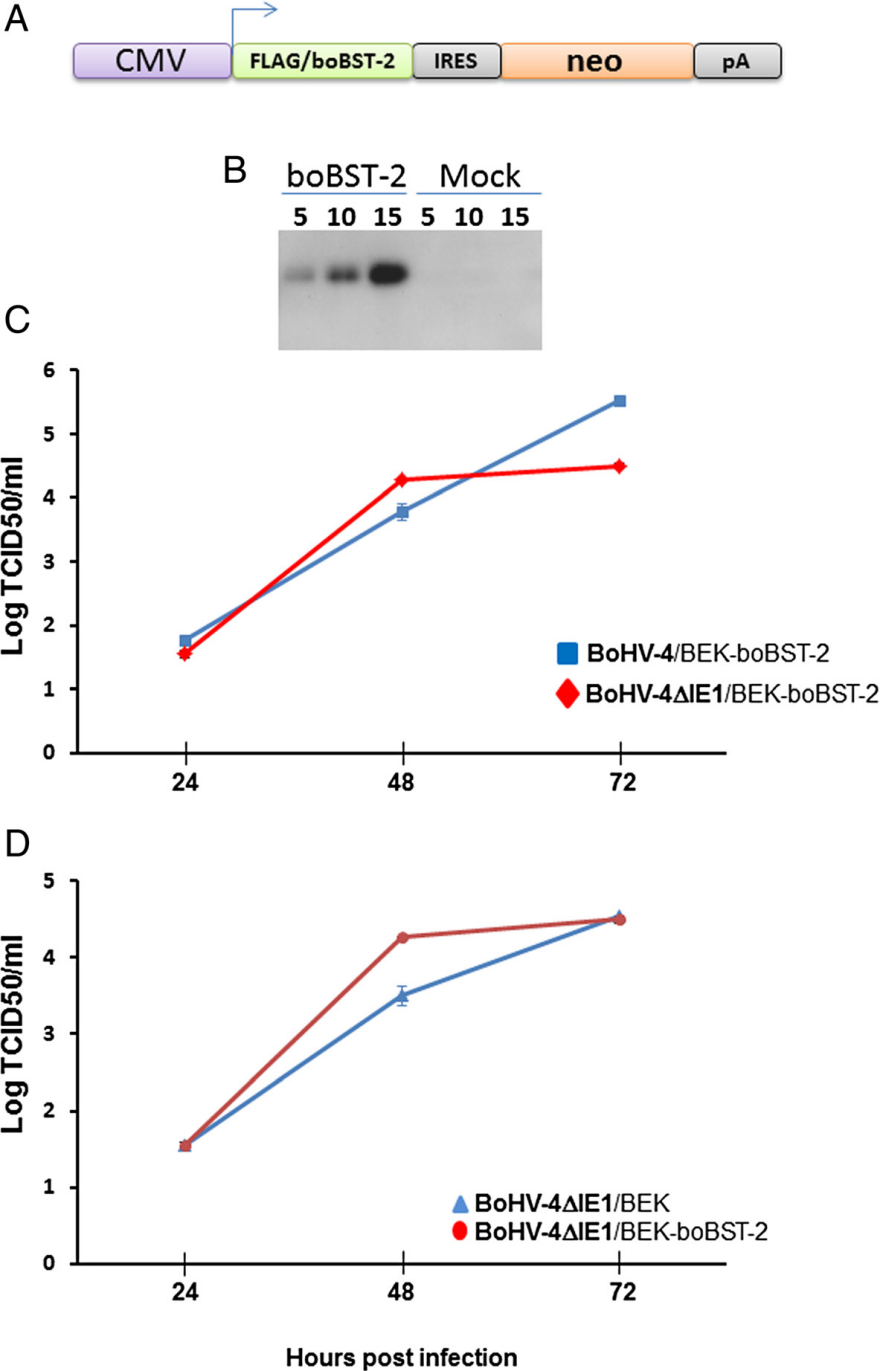
BoHV-4IE1 is not a tethering counteracting factor. **a** Schematic diagram (not on scale) of the bicistronic construct pCMV-boBST-2-IRES-*neo* containing the CMV promoter (*violet*), the flag tagged boBST-2 (*green*) ORF, an internal ribosomal entry site (IRES; *grey*), the neo ORF (*orange*) and a polyadenilation signal (pA; *grey*). **b** Western immunoblotting of BEK cells stably transfected with pCMV-boBST-2-IRES-*neo* (BEK-boBST-2 ) and selected with G418. The lanes were loaded with different amounts of total protein cell extract (5, 10 and 20 μg). Negative control was established with empty vector transfected cells (Mock). **c** Replication kinetics of BoHV-4ΔIE1 (*red line*) and BoHV-4 (*blue line*) on BEK-boBST-2 cells. **d** Replication kinetics of BoHV-4ΔIE1 made on BEK cells (*blue line*) and compared to that made on BEK-boBST-2 cells (*red line*)

Despite BoHV-4IE1 gene deletion gave a mild phenotype that could be rescued by BoHV-4IE1 gene expression *in trans*, the real function of BoHV-4IE1 gene, in the contest of viral replication, remained elusive. However from *in silico* study it was noticed that BoHV-4IE1 shares a conserved amino-terminal domain with the K5/K3-family gamma-2 herpesvirus plant homeo domain (PHD)/leukemia associated protein (LAP)-finger type [20]. In a proteomics screen for new host targets of KSHV K5, it was observed that bone marrow stromal cell antigen 2 (BST-2, also called tetherin), an interferon-induced protein with the unique ability to restrict the egress of some enveloped viruses [21], was counteracted in terms of viral egress restriction activity in the presence of K5 [22]. More recently, it was demonstrated that, similar to other K5 targets, BST-2 is ubiquitinated by K5, resulting in ubiquitin-mediated endocytosis and lysosomal destruction [23]. Starting from these information the potential BoHV-4IE1 counteracting properties toward BST-2 were investigated. For this purpose a cell line, BEK-boBST-2, over expressing bovine BST-2 (boBST-2) was generated by stably transfecting BEK cells with a bicistronic construct, pCMV-boBST-2-IRES-*neo*, delivering a FLAG tagged boBST-2 ORF (Fig. 4a). G418 selected BEK-boBST-2 cells stably overexpressed boBST-2, as shown by western immunoblotting with a monoclonal antibody directed against the FLAG tag (Fig. 4b). The over-expression of boBST-2 in BEK cells and the deletion of IE1 in BoHV-4 genome should amplify the BoHV-4ΔIE1 phenotype, in terms of restriction release of BoHV-4ΔIE1 IRVPs into the infected BEK-boBST-2 cells supernatant, if BoHV-4IE1 was a real BST-2 counteracting factor. Therefore, the replication kinetics of BoHV-4ΔIE1 and BoHV-4 were compared on BEK-boBST-2, BEK/IE1 and BEK cells. However no significant viral titer decrease at 72 hs post infection for BoHV-4ΔIE1 infected BEK-boBST-2 cells supernatant respect to that of BoHV-4 infected BEK-boBST-2 cells supernatant (Fig. 4c) was detected. This 1 log viral titer difference remained equal to that revealed for BoHV-4ΔIE1 and BoHV-4 infected BEK cells supernatants (Fig. 2b). Moreover, noteworthy a paradoxical significant increase of the viral titer, at 48 hs post infection, for BoHV-4ΔIE1 infected BEK-boBST-2 cells supernatant respect to the supernatant of BoHV-4ΔIE1 infected BEK cells (Fig. 4d) was observed. Although this last data was not further investigated, it could be assumed a role different than a BST-2 counteraction factor for BoHV-4IE1 gene product. On the other hand the similarities observed *in silico* between K5 and BoHV-4IE1 were very mild and limited to the amino-terminal portion of the proteins (Additional file 1). This could justify the lack of functional similarity in terms of BST-2 counteracting activity observed for IE1 and represents a classical demonstration that the *in silico* prediction should be always experimentally verified before to draw any conclusion.

## Conclusions

BoHV-4 has been suggested as a vector for gene delivery [15, 16, 19, 24–27]. Several molecular and biological characteristics for this virus include: absence of oncogenicity, ability to accommodate large amounts of foreign genetic material, ability to be maintained in an episomal state in dividing cells and capability to infect several cellular types derived from different animal species. Although the genome of BoHV-4 has been sequenced completely [9, 28] the functional importance of most of the genes remains unknown. Targeting genes and regions of gene expression regulation and the phenotypic analysis of the BoHV-4 mutants represents a powerful tool to study the direct and indirect interactions of genes and genomic elements in a biological context. The classification of equivalent phenotypes carrying different types of mutations will provide insight into functional networks and their constituting elements. Fewer than 75–80 ORFs have been characterized in BoHV-4, based on amino acids homology and genome location and most of the genes have no apparent homology to known viral or cellular genes. Essential genes are of particular importance and the inactivation of at least one of them is required to create replication-incompetent herpesvirus vectors for vaccination or gene therapy, while non-essential genes can be removed to gain space for insertion of therapeutic genes or generate attenuated vaccines, as it the case of IE1 investigated in the present work. Therefore, these data are extremely valuable when virus genomes, such as BoHV-4, are modified for vector purposes.

## Methods

### Cells

Bovine embryo kidney [(BS CL-94) BEK, from M. Ferrari, Istituto Zooprofilattico Sperimentale, Brescia, Italy], Madin Darby Bovine Kidney (MDBK, ATCC: CCL-22), African green monkey kidney epithelial cells [(VERO, ATCC: CCL-81) kindly provided by Professor S. Trees, University of Liverpool], BEK expressing cre recombinase (BEK*cre*) cells [16] and HEK [Human Embryo Kidney 293 T cells [(HEK 293 T) ATCC: CRL-11,268] were cultured in EMEM (Lonza) containing 10 % FBS, 2 mM L-glutamine (SIGMA), 100 IU/mL penicillin (SIGMA) and 100 mg/mL streptomycin (SIGMA) and 2.5 mg/mL amphotericin B.

### Plasmids generation and PCR reactions

pFlagbovTeth [29] was linearized with PvuI restriction enzyme digestion and used as a template to amplify flagged bovine tetherin ORF. The amplification was carried over 35 cycles, each cycle consisting of denaturation at 94 °C for 1 min, primer annealing at 55 °C for 1 min, and chain elongation with 2 U of Pfu DNA polymerase (Thermoscientific) at 72 °C for 40 s. PCR amplification was carried out in a final volume of 50 μL of 10 mM Tris–hydrochloride pH 8.3 containing 0.2 mM deoxynucleotide triphosphates, 3 mM MgCl_2_, 50 mM KCl, 5 % dimethyl sulfoxide (DMSO) and 0.25 μM of each primer [BST2 sense-EcoRV and BST2 –anti-NheI primers (Table 1)]. The ~600 bp PCR amplicon product was checked by sequencing and then subcloned in the expression vector pIres2neo (Clontech), cut with NheI/EcoRV, to generate pCMV-boBST-2-IRES-*neo*.

To generate pCMV-IE1-IRES-*neo,* BoHV-4IE1 ORF cDNA was amplified by PCR from reverse transcribed total RNA of BoHV-4 infected BEK cells, using the Ready to Go system (Amersham), according to the manufacturer’s instructions. The PCR was performed using IE1-NheI-sense and IE1-BamHI-antisense primers (see Table 1) over 35 cycles, each cycle consisting of denaturation at 94 °C for 1 min, primer annealing at 58 °C for 1 min, and chain elongation with 2 U of Pfu DNA polymerase (Thermoscientific) at 72 °C for 1 min. The amplification was carried out in a final volume of 50 μL of 10 mM Tris–hydrochloride pH 8.3 containing 0.2 mM deoxynucleotide triphosphates, 3 mM MgCl_2_, 50 mM KCl, 5 % DMSO and 0.25 μM of each primer. The 900 bp IE1 ORF cDNA amplicon was then cut with NheI/BamHI and inserted in an expression vector, pIres2neo (Clontech) cut with the same enzymes.

To amplify Immediate Early 1 homology arm regions one microgram of pBAC-BoHV-4 DNA sample was amplified over 35 cycles, each cycle consisting of denaturation at 94 °C for 1 min, primer annealing at 56 °C for 1 min, and chain elongation with 2 U of Pfu DNA polymerase (Thermoscientific) at 72 °C for 2 min. PCR amplification was carried out in a final volume of 50 μL of 10 mM Tris–hydrochloride pH 8.3 containing 0.2 mM deoxynucleotide triphosphates, 3 mM MgCl_2_, 50 mM KCl, 5 % DMSO and 0.25 μM of each primer. The primers used to amplify BoHV-4IE1 left and right arms, were IE1-left-sense and antisense (introducing a EcoRI site in the sense primer and a KpnI site in the antisense) and IE1-right-sense and antisense (inserting a PstI site in the sense primer and a HindIII in the antisense primer) (see Table 1). The so generated 938 and 992 bp amplicons (respectively for the left and the right arms) were then checked in 1 % agarose gel and visualized after ethidium bromide staining in 1 × TAE buffer (40 mM Tris-acetate, 1 mM EDTA) and used to subclone the IE1 left and right homology arm regions in an expression vector. The specificity of the PCR products were determined by sequencing.

The 938 and 992 bp BoHV-4IE1 left and right arm amplicons were firstly inserted in pJET1.2, a commercial vector from Thermoscientific, designed to directly subclone blunt-ended PCR amplicons (as those generated by Pfu polymerase amplification), using the CloneJet PCR Cloning Kit (Thermoscientific), generating pJET/IE1left and pJET/IE1 right.

pTZ-KanaGalK was generated by sub-cloning the 2232 bp galactokinase prokaryotic expression cassette (GalK), along with the kanamycin resistant expression cassette (Kana), into the pTZ57R/T shuttle vector, cut with KpnI/PstI [30]. The targeting vector, pIE1L-KanaGalK-IE1R, was generated firstly by the insertion of the 930 bp left IE1 arms, excised from pJET/IE1left, cut with EcoRI/KpnI, in pTZ-KanaGalK, cut with the same enzymes; in this intermediate construct, cut with PstI/HindIII, was consequently subcloned the 983 bp right IE1 arm, obtained from pJET/IE1right, cut with the same enzymes.

### Stable cell lines generation

BEK cells from a sub-confluent 75 cm^2^ flask were electroporated (Equibio apparatus; 270 V, 1500 μF) with 10 μg of pCMV-IE1-IRES-*neo* DNA in Dulbecco’s Modified Eagle Medium with high glucose (DMEM high) without FBS. Electroporated cells were transferred to new 75 cm^2^ flasks and fed with complete medium (EMEM containing 10 % FBS, 100 IU/mL of penicillin, 100 mg/mL streptomycin, 2.5 mg/mL amphotericin B, and 2 mM L-glutamine). Twenty-four hours after electroporation, stably transfected cells were selected with 700 μg/mL of G418 (Sigma) until visible colonies appeared on the surface of the flask. Some selected clones were independently growth for 20 passages in the presence of G418. Thus, BEK stably expressing IE1 cDNA, BEK/IE1, were obtained.

BEK cells from a sub-confluent 75 cm^2^ flask were also transfected through electroporation (Equibio apparatus; 270 V, 1500 μF) with 10 μg of pCMV-boBST-2-IRES-*neo* DNA in DMEM high in the absence of FBS. Electroporated cells were transferred to new 75 cm^2^ flasks containing complete medium. Twenty-four hours after electroporation, stably transfected cells were selected with 700 μg/mL of G418 (Sigma) until visible colonies appeared on the surface of the flask. The selective pressure of G418 was maintained during the passages and BEK stably expressing the flagged bovine tetherin cDNA, BEK-boBST-2, were generated.

### BAC recombineering and selection

Recombineering was performed as previously described [31] with some modifications. Five hundred microliters of a 32 °C overnight culture of SW102 containing BAC-BoHV-4 were diluted in 25 mL Luria–Bertani (LB) medium with or without chloramphenicol (SIGMA) selection (12.5 μg/mL) in a 50 mL baffled conical flask and grown at 32 °C in a shaking water bath to an OD_600_ of 0.6. Then, 12 mL were transferred to another baffled 50 mL conical flask and heat shocked at 42 °C for exactly 15 min in a shaking water bath. The remaining culture was left at 32 °C as the un-induced control. After 15 min the two samples, induced and un-induced, were briefly cooled in ice/water bath slurry and then transferred to two 15 mL Falcon tubes and pelleted using 5000 r.p.m. (Eppendorf centrifuge) at 0 °C for 5 min. The supernatant was poured off and the pellet was resuspended in 1 mL ice cold dH2O by gently swirling the tubes in ice/water bath slurry. Subsequently, 9 mL ice-cold dH2O were added and the samples pelleted again. This step was repeated once more, the supernatant was removed and the pellet (50 μL each) was kept on ice until electroporated with the gel-purified IE1L-KanaGalK-IE1R fragment obtained by linearizing pIE1L-KanaGalK-IE1R, with HindIII (Thermoscientific). An aliquot of 150 ng of the purified fragment was used for each electroporation in a 0.1 cm cuvette at 25 mF, 2.5 kV and 201 V. After electroporation, a recovery of 1 h of the bacteria in 1 mL of LB Broth (BD Biosciences) (15 mL Falcon tube) was performed in a 32 °C shaking water bath. After two washes in sterile M9 salt (6 g/L Na_2_HPO_4_, 3 g/L KH_2_PO_4_, 1 g/L NH_4_Cl, 0.5 g/L NaCl) (SIGMA), serial dilutions of the bacteria were plated on M63 minimal medium plates containing 15 g/L agar (Invitrogen), 0.2 % D-galactose (SIGMA), 1 mg/L D-biotin (SIGMA), 45 mg/L L-leucine (SIGMA) and 12.5 mg/mL chloramphenicol (SIGMA). Plates were incubated 3 days at 32 °C. Several selected colonies were picked up, streaked on McConkey agar indicator plates (DIFCO, BD Biosciences) containing 1 % D-galactose and 50 μg/mL of kanamycin and incubated at 32 °C for 3 days until red colonies appeared. Red colonies were grown in duplicate for 5–8 h in 1 mL of LB containing 50 mg/mL of kanamycin (SIGMA) or LB containing 12.5 mg/mL of chloramphenicol. Only those colonies growing both on chloramphenicol and on kanamycin were kept and grown overnight in 5 mL of LB containing 12.5 mg/mL of chloramphenicol. BAC DNA was purified and analyzed through restriction enzyme digestion for IE1L-KanaGalK-IE1R fragment targeted integration. Original detailed protocols for recombineering can also be found at the recombineering website (http://recombineering.ncifcrf.gov).

### Restriction enzyme analysis and non-isotopic Southern hybridization

Fifteen microliters of BAC DNA prepared from bacteria containing pBAC-BoHV-4 were restriction enzyme digested with PstI, separated by electrophoresis overnight in a 1 % agarose gel, stained with ethidium bromide, capillary transferred to a positively charged nylon membrane (Roche), and cross-linked by UV irradiation by standard procedures. The membrane was pre-hybridized in 50 mL of hybridization solution (7 % SDS, 0.5 M phosphate, pH 7.2) for 1 h at 65 °C in a rotating hybridization oven (Techna instruments). Probe preparation and digoxigenin non-isotopic labelling was performed by PCR. Southern Blotting probe was designed spanning Kana region using the primer pair SmaI-AseI-Kana sense and SmaI-MluI-Kana anti (see Table 1). PCR amplification was carried out in a final volume of 50 μL of 10 mM Tris–HCl, pH 8.3, containing 0.2 mM deoxynucleotide triphosphates, 0.02 mM alkaline labile digoxigenin-dUTP (deoxyuridine triphosphate) (Roche), 3 mM MgCl_2_, 50 mM KCl, and 0.25 μM of each primer over 35 cycles, each cycle consisting of denaturation at 94 °C for 1 min, primer annealing at 56 °C for 1 min, and chain elongation with 1 U of Taq polymerase (Thermoscientific) at 72 °C for 1 min. Five microliters of the probe were added to 500 μL of dH_2_O into a screw-cap tube, denatured in boiling water for 5 min, and cooled down on ice for another 2 min. Denatured probe was added to 50 mL of pre-heated 65 °C hybridization solution [7 % SDS, 0.5 M phosphate, pH 7.2 and 1 mM ethylenediaminetetraacetic acid (EDTA)] to the pre-hybridized membrane and hybridized overnight at 65 °C in a rotating hybridization oven (Techna Instruments). Following hybridization, the membrane was washed twice for 30 min with 100 mL of washing solution I (0.5× SSC [1× SSC is 0.15 M NaCl plus 0.015 M sodium citrate] and 0.1 % SDS) and twice for 30 min with 100 mL of washing solution II (40 mM phosphate, pH 7.2, 0.05 % SDS) at 65 °C. On a freshly washed dish, the membrane was incubated for 30 min at room temperature in 100 mL of blocking solution (100 mM maleic acid, pH 7.5, 150 mM NaCl, 1 % blocking reagent [Roche]). Anti-digoxigenin Fab fragment (150 U/200 μL [Roche]), diluted 1:15.000 in 50 mL of blocking solution, was applied to the membrane for 30 min under gentle shaking at room temperature and washed twice for 15 min with 100 mL of washing solution (100 mM maleic acid, pH 7.5, 150 mM NaCl, 0.3 % Tween 20). Detection was performed following equilibration of the membrane in detection buffer (100 mM Tris–HCl, pH 9.5, 1 mM EDTA) for 2 min at room temperature. Chemiluminescent substrate (CSPD, Roche) was added by scattering the drops over the surface of the membrane. Signal detection was obtained, exposing the membrane to X-ray film. The exposure time was adjusted with the intensity of the signal.

### Cell culture electroporation and recombinant virus reconstitution

Plasmid BAC DNAs (5 μg) in 600 μL DMEM without serum were electroporated (Equibio apparatus, 270 V, 960 μF, 4-mm gap cuvettes) into BEK or BEK*cre* cells from a confluent 25 cm2 flask. Electroporated cells were then returned to the flask, fed the next day, and split 1:2 when they reached confluence at 2 days post electroporation. Cells were left to grow until Cytopathic Effect (CPE) appeared. Recombinant viruses were propagated by infecting confluent monolayers of BEK and MDBK cells at a multiplicity of infection (m.o.i.) of 0.5 50 % tissue culture infectious doses (TCID50) per cell and maintaining them in MEM with 10 % fetal bovine serum (FBS) for 2 hs. The medium was removed and replaced by fresh MEM containing 10 % FBS. When approximately 90 % of the cell monolayer exhibited CPE (~72 h post infection), the virus was prepared by freezing and thawing cells three times and pelleting virions through 30 % sucrose, as described previously [16]. Virus pellets were resuspended in cold MEM without FBS. TCID50 were determined on MDBK or BEK cells by limiting dilution.

### Viruses and viral replication

Bovine herpesvirus-4 (BoHV-4), BoHV-4ΔTK [15] and BoHV-4ΔIE1, were propagated by infecting confluent monolayers of VERO, BEK, BEK-IE1 and BEK-boBST2 cells at a multiplicity of infection (m.o.i.) of 0.5 50 % tissue culture infectious doses (TCID50) per cell and maintained in MEM with 10 % FBS for 2 hs. The medium was then removed and replaced by fresh MEM containing 10 % FBS. When approximately 90 % of the cell monolayer exhibited cytopathic effect (CPE) (approximately 72 hs post-infection), the virus was prepared by freezing and thawing cells three times and pelleting the virions through 30 % sucrose, as described above. Virus pellets were resuspended in cold MEM without FBS. TCID50 were determined in BEK or MDBK cells by limiting dilution. Viruses growth kinetics were assessed infecting BEK, BEK-IE1 and BEK-boBST2 cells with 0.1 m.o.i. of virus, cells were monitored periodically for 72 hs at the microscope to verify the CPE appearance and small aliquots of medium were recovered at 24, 48 and 72 hs and titrated in BEK cells. Viral titer differences between each time point are the averages of triplicate measurements ± standard errors of the means and were analyzed by analysis of variance (ANOVA).

### Viral plaque assay

VERO cell monolayers were infected with BoHV-4 and BoHV-4ΔIE1 with serial 10-fold dilutions of the viruses and incubated at 37 °C for 2 hs. Infected cells were then overlaid with fresh 2X EMEM (4 mM L-glutamine, 200 IU/mL penicillin, 200 mg/mL streptomycin, 5 mg/mL amphotericin B) supplemented with 12 % FBS and 0,6 % of carboxymethyl-cellulose. Cells were incubated at 37 °C with a 5 % CO^2^ atmosphere for 5 ds until the plaques were visible. The medium was discarded and, after a wash with sterile phosphate buffer saline (PBS), the cellular monostrates were fixed in 4 % Paraformaldehyde for 15 min at room temperature, and stained with 0,5 % crystal violet in absolute ethanol for 5–10 min at room temperature. Cells were washed with tap water and left to dry at room temperature. The diameter of the plaques was measured through a software for image analysis (AxioVision Rel. 4.6. digital image processing software; Carl Zeiss).

### Western immunoblotting

Cell extracts were obtained from BEK-boBST2 scraped from 25 cm^2^ confluent flasks at several different passage levels (from 2nd to 10th) by adding 100 μL of cell extraction buffer (50 mM Tris–HCl, 150 mM NaCl, and 1 % NP-40; pH 8) to cell pellets. Cell extracts containing 50 μg of total protein were electrophoresed through sodium dodecyl sulfate-10 % polyacrylamide gels and transferred to nylon membranes by electroblotting. Membranes were incubated with mouse anti-FLAG M2 monoclonal antibody (1:1000 dilution; F1804; SIGMA), which was detected with horseradish peroxidase-labelled goat anti rabbit immunoglobulin G1 (IgG1) antibody (1:15.000; A0545; Sigma), and visualized by enhanced chemiluminescence (ECL Kit; Pierce).

### Statistics

Data were analyzed using one way analysis of variance (ANOVA) followed by Dunnet’s post hoc test for group comparisons. Results are reported as mean ± SD and significance was attributed when P,0.05 (*) or P,0.01 (**).

## Additional file

Additional file 1:
**Alignment between KSHV K5 and BoHV-4IE1 protein sequence.** The sequence of KSHV K5 protein was aligned with BoHV-4 Immediate early 1 gene product to highlight the percentage of sequence identities and positive matches through the alignment software BLAST-2 (https://blast.ncbi.nlm.nih.gov/Blast.cgi?PROGRAM=blastp&PAGE_TYPE=BlastSearch&BLAST_SPEC=blast2seq&LINK_LOC=blasttab). (TIFF 5192 kb)

## Abbreviations

VERO: African green monkey kidney epithelial cells
aa: Amino acids
ANOVA: Analysis of variance
BAC: Bacterial artificial chromosome
BEK: Bovine embryo kidney
BEK-boBST-2: BEK expressing bovine tetherin cDNA
BEK*cre*: BEK expressing cre recombinase
BST-2: Bone marrow stromal cell antigen 2
boBST-2: Bovine BST-2
BoHV-4: Bovine herpesvirus 4
CMV: Cytomegalovirus
CPE: Cytopathic effect
DMSO: Dimethyl sulfoxide
DMEM: Dulbecco’s modified eagle medium
dUTP: Deoxyuridine triphosphate
E: Early genes
EDTA: Ethylenediaminetetraacetic acid
EMEM: Eagle’s minimum essential medium
FBS: Fetal bovine serum
GalK: Galactokinase
HEK 293 T: Human embryo kidney 293 T cells
IE: Immediate early genes
IgG1: Immunoglobulin G1
IRVPs: Infectious replicating viral particles
IRES: Internal ribosomal entry site
KSHV: Kaposi sarcoma associated herpesvirus
L: Late genes
LAP: Leukemia associated protein
LB: Luria–Bertani medium
MDBK: Madin darby bovine kidney
m.o.i: Multiplicity of Infection
ORFs: Open reading frames
PBS: Phosphate buffer saline
PHD: Plant homeo domain
SDS: Sodium dodecyl sulfate
TAE: TrisAcetate buffer
TK: Thymidine kinase gene
TCID50: Tissue culture infectious doses_50_
