# Supplementary figures and images for: BoHV-4 immediate early 1 gene is a dispensable gene and its product is not a bone marrow stromal cell antigen 2 counteracting factor
Source: BMC Vet Res. 2015 Aug 27;11:224. doi: 10.1186/s12917-015-0540-4 (PMC4549876; doi:10.1186/s12917-015-0540-4)

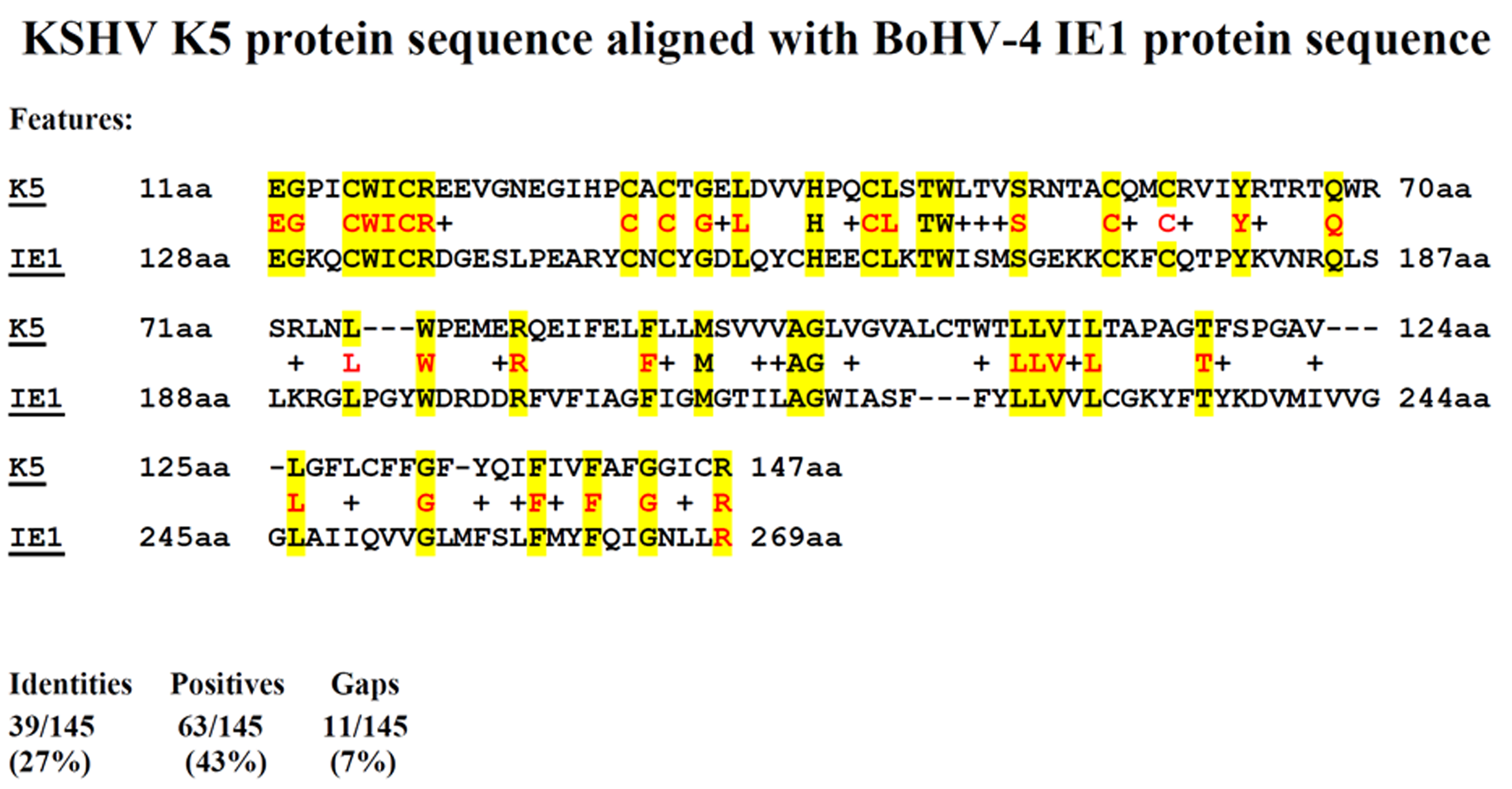

Supplement: Additional file 1: — Alignment between KSHV K5 and BoHV-4IE1 protein sequence. The sequence of KSHV K5 protein was aligned with BoHV-4 Immediate early 1 gene product to highlight the percentage of sequence identities and positive matches through the alignment software BLAST-2 (https://blast.ncbi.nlm.nih.gov/Blast.cgi?PROGRAM=blastp&PAGE_TYPE=BlastSearch&BLAST_SPEC=blast2seq&LINK_LOC=blasttab). (TIFF 5192 kb) [file 12917_2015_540_MOESM1_ESM.tif]
